# POU2AF2/OCA-T1 coactivates POU2F2 and defines a lineage-specific dependency in diffuse large B-cell lymphoma

**DOI:** 10.64898/2026.08.30.748132

**Published:** 2026-09-01

**Authors:** Rima Tulaiha, Liam Shanley, Amanda Luvisotto, Ping Wang, Vipul Shukla, Zibo Zhao, Feng Yue, Ali Shilatifard, Christopher R Vakoc, Lu Wang

## Abstract

Lineage-restricted transcriptional programs establish cell identity and can create selective dependencies in cancer. Here, we identify POU2AF2, encoding the transcriptional co-activator OCA-T1, as a critical lineage-specific dependency in a subset of diffuse large B-cell lymphoma (DLBCL). Pan-cancer dependency analyses and patient cohorts reveal elevated POU2AF2 expression in genetically aggressive DLBCL, where its depletion markedly suppresses tumor growth *in vitro* and *in vivo* . Mechanistically, POU2AF2 cooperates with the B-cell lineage– defining transcription factor POU2F2 (OCT2) to activate lymphocyte activation gene programs through direct chromatin engagement, thereby sustaining malignant transcriptional networks. We further identified a key epigenetic regulatory axis composed of the lineage-specific transcription factor TCF3 and the histone methyltransferase SET1A-COMPASS that drives POU2AF2 expression downstream of B-cell receptor signaling. Single-cell transcriptomic analysis reveals that POU2AF2 marks and sustains an innate-like B1 B-cell population *in vivo* , a candidate cell of origin for lymphoma. Together, these findings define a lineage-restricted POU2AF2/POU2F2 transcriptional module, controlled by a TCF3/SET1A epigenetic network, that sustains both innate-like B-cell identity and malignant fitness in DLBCL. Our study uncovers a previously unrecognized lineage-specific transcriptional dependency and highlights POU2AF2 and its associated regulatory circuitry as potential therapeutic targets in aggressive B-cell malignancies.

## Full Text Availability

The license terms selected by the author(s) for this preprint version do not permit archiving in PMC. The full text is available from the preprint server.

